# Interdependences between finger movement direction and haptic perception of oriented textures

**DOI:** 10.1371/journal.pone.0208988

**Published:** 2018-12-14

**Authors:** Alexandra Lezkan, Knut Drewing

**Affiliations:** Department of General Psychology, Justus-Liebig University, Giessen, Germany; Pennsylvania State University, UNITED STATES

## Abstract

Although the natural haptic perception of textures includes active finger movements, it is unclear how closely perception and movements are linked. Here we investigated this question using oriented textures. Textures that are composed of periodically repeating grooves have a clear orientation defined by the grooves. The direction of finger movement relative to texture orientation determines the availability of temporal cues to the spatial period of the texture. These cues are absent during movements directed in line with texture orientation, whereas movements orthogonal to texture orientation maximize the temporal frequency of stimulation. This may optimize temporal cues. In Experiment 1 we tested whether texture perception gets more precise the more orthogonal the movement direction is to the texture. We systematically varied the movement direction within a 2IFC spatial period discrimination task. As expected, perception was more precise (lower discrimination thresholds) when finger movements were directed closer towards the texture orthogonal as compared to in parallel to the texture. In Experiment 2 we investigated whether people adjust movement directions to the texture orthogonal in free exploration. We recorded movement directions during free exploration of standard and comparison gratings. The standard gratings were clearly oriented. The comparison gratings did not have a clear orientation defined by grooves. Participants adjusted movement directions to the texture orthogonal only for clearly oriented textures (standards). The adjustment to texture orthogonal was present in the final movement but not in the first movement. This suggests that movement adjustment is based on sensory signals for texture orientation that were gathered over the course of exploration. In Experiment 3 we assessed whether the perception of texture orientation and movement adjustments are based on shared sensory signals. We determined perceptual thresholds for orientation discrimination and computed ‘movometric’ thresholds from the stroke-by-stroke adjustment of movement direction. Perception and movements were influenced by a common factor, the spatial period, suggesting that the same sensory signals for texture orientation contribute to both. We conclude that people optimize texture perception by adjusting their movements in directions that maximize temporal cue frequency. Adjustments are performed on the basis of sensory signals that are also used for perception.

## Introduction

Imagine entering a room with the lights turned off. In order to perceive the world around you with your sense of touch, you would probably move your hands and explore. The way you would move your hands will depend on the things you encounter. In other words, hand movements generate haptic sensations [1] and exploratory movements depend on the object property of interest [2]. Recent studies described the mutual influence of movement and sensation in haptic perception of location, softness, and roughness [3–5]. Our study investigates the interaction between movement and sensation in natural exploration of oriented texture. In three experiments, we test whether people optimize the perception of oriented textures by adjusting the direction of exploratory movements based on sensory signals for texture orientation. Experiment 1 tests whether there is a systematic influence of movement direction on the precision of perceiving the spatial period of oriented textures. Experiments 2 and 3 investigate whether sensory signals for texture orientation influence the control of movement directions, first by studying whether movement direction is adjusted to the orientation of the explored texture (Experiment 2), and then by investigating whether sensory signals that underlie the perception of texture orientation are also used in the adjustment of movement direction (Experiment 3).

Texture perception by touch is multidimensional [6] and people can describe multiple facets of a surface texture including roughness, coarseness, jaggedness, spatial element density, or configuration [7]. However, texture perception has often been investigated using rather simple textures such as periodic grooved gratings, which can be defined by their spatial period (e.g., [8]), and most researchers have asked for roughness judgments (e.g., [9–11]). However, several others also asked for a more direct spatial period judgment (e.g., [12–15]). Results from both tasks suggest that haptic perception of such aspects of the structure of textures is based on spatial and temporal cues [13,16]. Spatial cues are the kind of information we can get from skin deformation after pressing a textured surface against the skin without permitting lateral movement (e.g., [17]). The neural coding of spatial cues, as shown in roughness perception, is strongly based on the spatial pattern of activation of the slowly adapting afferents (SA1) [16]. Temporal cues arise from movement over a textured surface and refer to the changes of signals over time, i.e. vibrations (e.g., [18]). Those vibrations are mainly coded by rapidly adapting (RA) and Pacinian (PC) afferents, as also shown in the perception of roughness [16]. Although, in natural situations, textures are typically explored with lateral movements [2], it has been previously discussed how much movements can actually enhance perceptual precision (at least for certain kind of textures) [19–20]. For fine textures, movements seem to be crucial; roughness discrimination was reported to be seriously impaired without the temporal cues produced by movements [21]. In contrast, the roughness of coarse textures was reported to be highly distinguishable by static touch alone [17]. Nevertheless, there is evidence that even for coarse textures, as well as for most natural surfaces, spatial cues are combined with temporal cues [9,16]. Gamzu and Ahissar [13] demonstrated the advantage of temporal cues. For their frequency (= 1/spatial period) discrimination task, poor haptic performers were able to improve by changing movement velocity as a strategy, which accentuated temporal cues. Similarly, Lamb [22] showed that when exploration generates temporal cues, the precision of texture perception can be increased. In his study, textures, which incorporated stripes of raised dots, were passively moved against the participant’s finger. The spacing between stripes was either modified perpendicular to the movement track or along the movement track. After the sequential presentation of two textures, participants reported in which of the two textures the spacing between stripes was modified. Performance was better for manipulations along the movement track than perpendicular to it. This can be attributed to the added temporal cues in the case of variations along the movement track. These two reported studies indicate that not only movements (or the lack of them) but also the specific movement parameters matter. More precisely, the study of Gamzu and Ahissar describes an influence of movement velocity on perception and Lamb’s study describes an influence of direction of passive movement between the skin and the surface. For oriented textures, movement direction is systematically linked to temporal cue frequency. Therefore, if temporal cues matter in active touch, movement direction should impact the perception of the spatial period of the texture. To our knowledge, however, there exists no study that investigated the influence of movement direction in active touch on perceptual precision and did so by systematically varying movement direction.

Assuming that there is one movement direction that leads to the best perceptual precision, it can be referred to as the optimal movement direction. But do humans utilize this optimal movement direction in free exploration? Freely chosen movements used in active exploration were suggested to aim for maximization of sensory information gain (e.g., [23–24]). As a matter of fact, in visual research, the orientation of depicted textures was found to influence eye movement direction [25–27]. For haptic softness and shape perception, it has been demonstrated that participants enhance the precision of perception through motor control [28–29]. For roughness perception, Tanaka, Bergman Tiest, Kappers, and Sano [5] observed that participants adjust normal force, scanning velocity, and break times in ways that seem effective for different tasks and explored stimuli. Along these lines, Nefs, Kappers and Koenderink [14] reported that applied contact force increased with line frequency of gratings and suggested that this might have improved perception in the task. However, these two studies on texture perception have not assessed whether movement adjustments actually optimize perceptual precision, neither did they investigate movement direction.

The objective of the current study is to investigate the interdependence between sensation and movements in the perception of texture spatial period. Our hypothesis is that humans adjust their movement direction when exploring oriented textures in order to optimize perceptual performance, and that they do so based on sensory signals for texture orientation. Our textures are defined by periodic parallel grooves; they are orientated by the groove orientation. For these oriented textures, movement direction and temporal cues are systematically linked. Finger movements in the direction of the texture orientation do not produce temporal cues to the spatial period of the texture. Finger movements directed orthogonally to the texture orientation produce temporal cues with maximal frequency. The more movement directions are shifted from the texture orthogonal (i.e., the direction along which a grating modulates), the lower is the temporal frequency of stimulation. Therefore, the temporal frequency also differs less between textures with different spatial frequencies, which probably yields less precise estimates of spatial frequency. By prescribing the movement direction on oriented textures, Experiment 1 systematically investigates the impact of movement direction on the perception of spatial period of textures. We expect that perceptual precision is enhanced when movements are directed orthogonally to the texture. Experiments 2 and 3 test whether participants use sensory signals for texture orientation in order to optimize movement directions.

In Experiment 2, we investigate adjustments of movement direction over different strokes of the exploration process. Any adjustment of movement direction can only be based on the sensory signals gathered during the exploration process, when no prior knowledge is given. Thus, movement direction will only be adjusted after sufficient sensory signals for texture orientation are available. The integration of sensory signals can extend over several movements [30], and, because sensory signals are accumulated haptic perception becomes more precise with extended exploration [31]. Hence, we expect that only in the late strokes, at the end of natural exploration movement, are directions adjusted to optimize temporal cues (i.e., towards the texture orthogonal). Note that a previous analysis of part of the data of Experiment 2 has been pre-published in a conference paper [32]. However, here a considerably improved analysis of movement data has been used, so that the present results have not been previously published.

In Experiment 3, we investigate whether adjustments of movement direction and the perception of texture orientation rely on a common basis, namely shared sensory signals for texture orientation. In vision, numerous studies have investigated how far underlying sensory signals are shared by eye movement control and perception. These studies compared perceptual precision to eye movement precision as derived from psychophysical and ‘oculometric’ functions, respectively (e.g., [33–35]). Here, we construct ‘movometric’ functions based on the exploratory behavior, which allow for the direct comparison between the precision of motor adjustments and the perception of texture orientation. We expect that the precision of perception and movement vary with the same factor, namely texture period.

## Experiment 1

Experiment 1 investigates the impact of movement direction on the perception of texture period. Haptic texture stimuli were 3D printed (Stratasys Objet 30 Pro). All gratings were cylindrical discs with a groove pattern following a sine-wave function on top of the surface. Participants stroked once over each of two gratings in a pair and judged which one had a higher spatial frequency (= 1/spatial period). We used a PHANToM force-feedback device to restrict finger movements to specific directions by defining exploration tunnels (orientation: 0°, 30°, or 60°). The movement direction relative to the texture orthogonal was manipulated (0° vs. 45° vs. 90° shifted from the texture orthogonal). The orientation of the textures relative to the body was varied systematically depending on the exploration tunnel orientation and the movement direction relative to the texture orthogonal. For each of the relative movement directions we measured the just noticeable difference (JND) of the textures’ spatial period. Based on the decreasing availability of temporal cues, we predict a systematic increase in JNDs (i.e., discrimination thresholds assessing perceptual precision) with higher shifts from orthogonal movement direction.

### Methods and materials

#### Participants

The sample was composed of sixteen right-handed participants aged 19–29 years (11 females). All participants were naïve to the purpose of the experiment and were paid for participating. Nobody reported recent injuries of the right index finger or sensory or motor impairments. All had a two-point discrimination threshold of 3 mm or lower at the finger pad of the right index finger. In all three experiments, the reported methods and procedures were approved by the local ethics committee (LEK) of FB 06 at Giessen University (approval number: 2013–0021). Participants gave written informed consent. The study was conducted in accordance with the ethical standards laid down in the 2008 Declaration of Helsinki.

#### Apparatus and stimuli

**Fig 1:**
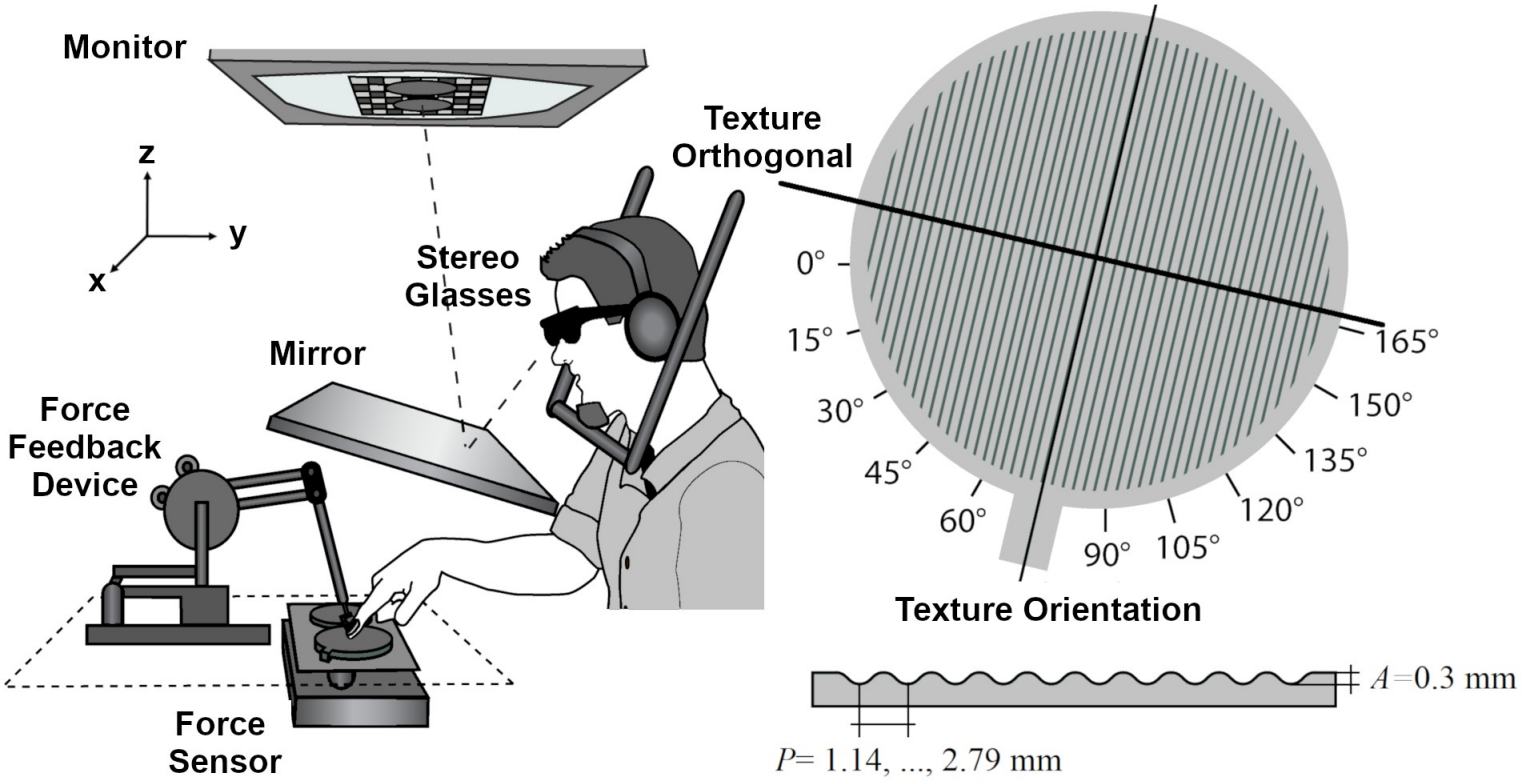
Sketch of setup and stimulus. Stimulus location, shape and the exploration tunnel contour were visually represented on a monitor and were seen through a mirror and stereo glasses. The participant’s right index finger was connected to the PHANToM via an adapter. The PHANToM measured the finger position and restricted the movement to a predefined exploration tunnel. Both real grating stimuli were placed in the same orientation next to each other on a force sensor.

Participants sat in front of a visuo-haptic setup (see Fig 1). The setup contained a PHANToM 1.5A haptic force feedback device, force sensor (682 Hz, resolution: 0.05 N) and a 22"-computer screen (120 Hz, 1024 x 1280 pixel). Circular grating stimuli were presented next to each other placed on the force sensor, which measured the finger force applied to the stimuli. Participants looked on the computer screen through stereo glasses and a mirror (40 cm viewing distance in total). Due to this mirror, participants were not able to see the real stimuli or their hand. Additionally, the setup allowed for a spatial alignment of the 3D-visual representation with the haptic display. In the virtual 3D-scene stimuli were displayed as three dimensional cylindrical discs with a border. This visual representation did not present the texture pattern or its orientation. The participant’s finger position was visible as a small sphere (8 mm diameter) when moving outside the stimulus area. We connected the right index finger to the PHANToM via an adapter, which was attached by double-faced adhesive tape to the nail. This setup allowed for free finger movements having all six degrees of freedom in a 38 x 27 x 20 cm³ workspace. However, here we used the PHANToM device to restrict finger movements to follow a predefined direction within the exploration tunnel and to measure finger position. Exploration tunnels were defined by a 16 mm wide path across the texture’s surface, where the PHANToM device displayed no force. Outside this exploration tunnel, forces *F* (in *N*) were presented that drove the finger back to the exploration tunnel, and increased by a square function with the finger’s distance *D* (in *mm*) to the tunnel’s border ($F=\sqrt{2}D²/441\;mm^{2}/N$). The exploration tunnel was displayed by a cuboid on top of the stimulus in the 3D-visual representation. In order to provide stable 3D vision, the participants head was stabilized by a chinrest. Custom-made software controlled the experiment, collected responses, and recorded the data from the force sensor and the PHANToM with recording intervals of 3 ms. We used headphones and ear plugs to mask sounds from haptic exploration.

Haptic gratings were created with the OpenSCAD software and 3D printing. The 3D printer (Objet 30 Pro, Stratasys Ltd., United States) builds drop-wise arbitrary 3D objects from 3D digital data (photopolymer material: VeroClear; build resolution: 600 x 600 x 1600 dpi (x-, y-, z-axis)). The grating discs were 4 mm high (z-axis) with a texture diameter of 90.7 mm (total diameter with border: 100.7 mm). A grip (10 x 5 mm) indicated the texture orientation for the experimenter (Fig 1). The height of the texture *z* followed a sine-wave function with the peak amplitude (*A*) of 0.3 mm (see Eq 1). The advantage of sine-wave stimuli is that they consist of only one spatial frequency component [36]. The standard stimulus had a period (*P*) of 1.78 mm. We created 25 comparison gratings with periods between 1.14 and 2.79 mm, with an approximate step size of 0.016*log (*P*). The spatial period of grooves was chosen so that they would be big enough to fall in the range of macrostructures (≥ 1mm) and small enough to lie in the range where manipulations of spatial period are in a monotonic relationship to perceived roughness (≤ 3 mm) [37,11].

$$z=\frac{1}{2}A\;sin\frac{2\pi x}{P}+\frac{1}{2}A$$ (1)

#### Design and procedure

Participants explored a stimulus pair consisting of one standard and one comparison stimulus in each trial. They judged which of the two had a higher spatial frequency, as this is more intuitive to judge than the spatial period. We explained spatial frequency as the number of experienced (i.e., felt) grooves over a certain distance. Note that textures included 40–80 ridges that were typically explored within less than a second (movement speed ~ 10 cm/s); therefore, counting of individual ridges is likely impossible. Stimuli with a longer period have lower spatial frequencies. We randomized which of the two stimuli was presented on the left side. During each trial, both stimuli of the stimulus pair were placed in the same orientation (example for one stimulus in Fig 1). The orientation of the stimulus pair was determined by a) the variable orientation of the exploration tunnel which was randomly chosen to be 0°, 30°, or 60°, and b) the presented level of the within-participant variable shift of movement direction from the texture orthogonal (0° vs. 45° vs. 90°). We measured just noticeable differences (JNDs) in terms of the discrimination of spatial period as a function of movement direction shift from the texture orthogonal. The lower the JNDs the better discrimination performance, that is to say the higher the perceptual precision. JNDs were assessed by the 75% discrimination threshold using the Best PEST adaptive staircase procedure [38] combined with the two-interval forced-choice task. In this method, the next comparison stimulus is chosen by an algorithm, which takes in to account previous responses for this condition. More precisely, the algorithm chooses the comparison with the maximum likelihood of being the threshold. In this way, the information gain in each step is maximized, which makes this method optimal in order to fasten threshold determination. The procedure came to an end after 45 trials per staircase. The final maximum-likelihood estimate in each staircase estimated the JND. For each condition, one upper and one lower staircase were implemented starting with the 2.79 and 1.14 mm, respectively. The trials from all 6 staircases were randomly interleaved. In order to practice the task and the movement restrictions through the exploration tunnel, participants performed 4 trials of each staircase prior to the experiment.

At the beginning of each trial a blank three dimensional cylindrical disc with a border indicated the location of the first stimulus (randomly assigned to the left and the right stimulus of the stimulus pair). Additionally, a cuboid on top of the stimulus displayed the exploration tunnel (orientation: 0°, 30°, or 60°). A dot, which was randomly assigned to be either on the left end (0°, 30°, or 60°) or the right end (180°, 210°, or 240°) of the exploration tunnel, indicated on which point the exploration should start. The visualization served to guide the participant through the trial without giving any information about textural structure or texture orientation. Participants were instructed to move from one point on the stimulus border to another through this ‘tunnel’ and they couldn’t see their hands moving during this time. After the participant stroke once over the texture within the exploration tunnel, the visualization of the second stimulus appeared. Exploration tunnel, shift from orthogonal, and starting point were identical for both stimuli of a pair. After one stroke over each stimulus, participants decided which of the two textures had a higher spatial frequency by pressing virtual buttons rendered by the PHANToM.

#### Data analyses

The data for each participant consisted of upper and lower JNDs for each of the three movement direction shifts from the texture orthogonal. In order to calculate JNDs for each movement direction shift, we averaged the corresponding upper and lower JND estimates. These values were entered into an ANOVA with the within-participant variable, Direction Shift of movement from the texture orthogonal (0° vs. 45° vs. 90°). We tested our hypothesis of a systematic monotonic increase in JNDs with higher Direction Shift by performing a linear contrast analysis on the direction-specific JNDs. Further, we calculated planned paired one-sided *t*-tests to analyze whether the contrasts between individual conditions reflect the increase in JNDs with higher Directional Shift.

Additionally, in order to check for the manipulation of the exploration tunnel, we analyzed the exploratory movement data. We extracted the direction of one stroke over each stimulus within each trial and averaged over the two strokes of a trial. Strokes were analyzed from exploratory parts of the movement, when the finger was touching the stimulus area with at least 0.1N of force for at least 200 msec. We detected strokes as continuous movements either from one texture border to another or between two movement turns, which we extracted by zero crossings in the 1st order derivatives of the x- or y-position over time. Stroke detection algorithms were considerably improved in comparison to a previous conference article on Exp. 2 [32], as follows: First, in order to exclude that curved movements will be detected as movement turns, we only included those zero crossings for which the 1st order derivative changed by more than 0.01 rad. Second, we increased the precision of measuring movement endpoints: In case the z-position of a movement turn was an outlier based on the exploratory part of the movements for this trial, stroke endpoints were defined as the closest positions within the 95%-confidence interval of z-positions. In case several strokes over one stimulus were detected by the algorithm (which might occur due to movement pause or slip) we analyzed only the stroke with the longest duration. We included only trials in this analysis for which we were able to extract strokes from the movement data for both stimuli of a trial (94%).

### Results

#### Movement data

**Fig 2:**
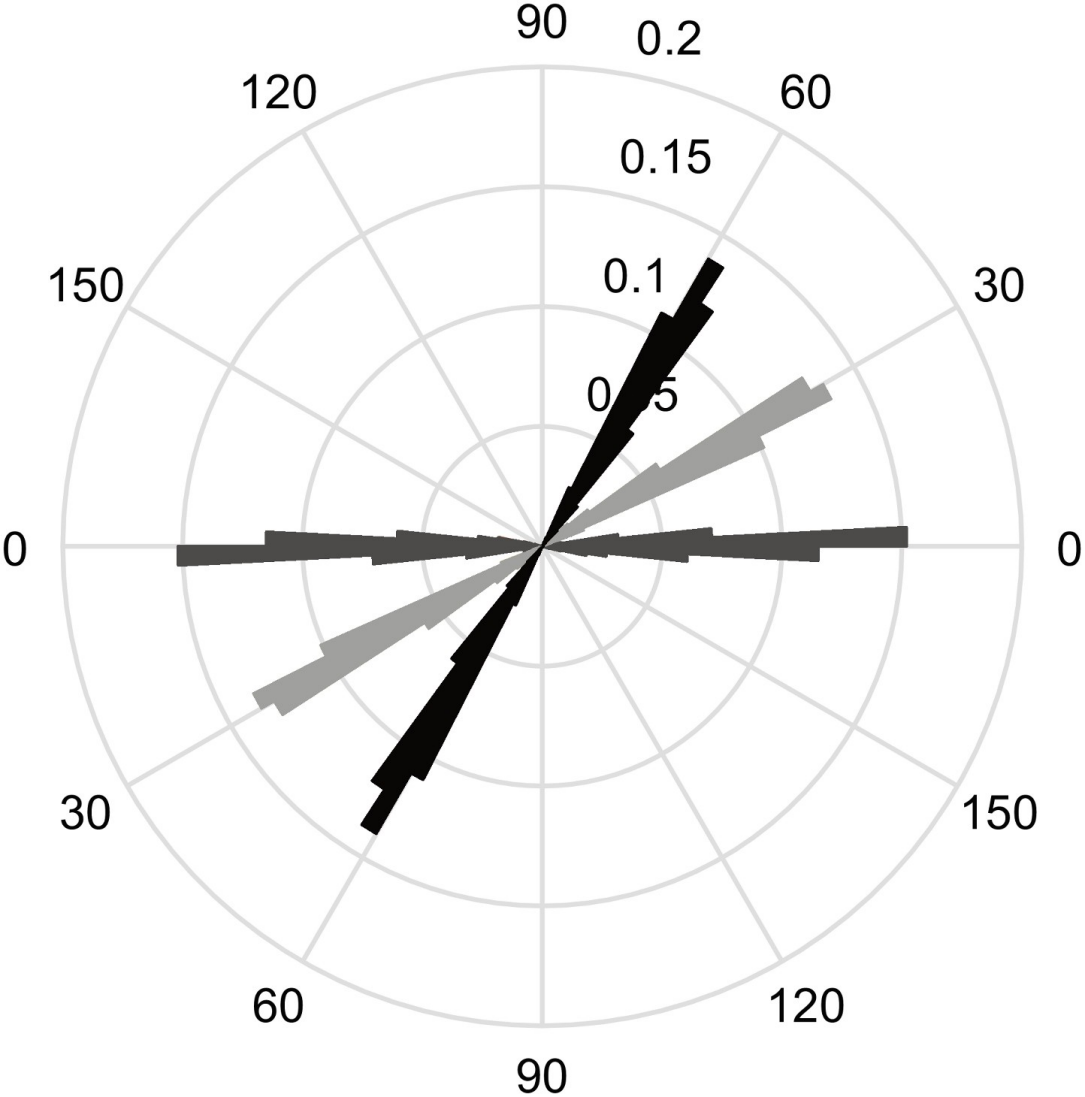
Movement directions. Circular histogram (bin size 3°) of all trials and participants for different exploration tunnels. Movement direction for trials with the exploration tunnel of 0° are plotted in dark gray, light grey stands for the 30°, and black for the 60° exploration tunnel. The numbers indicate the proportion of strokes in a certain direction.

We plotted the movement directions from all participants and all trials in a circular histogram (see Fig 2). The different colors represent trials with different exploration tunnels. As can be seen from the graph, movements followed the aimed direction with little spread.

#### JNDs

**Fig 3:**
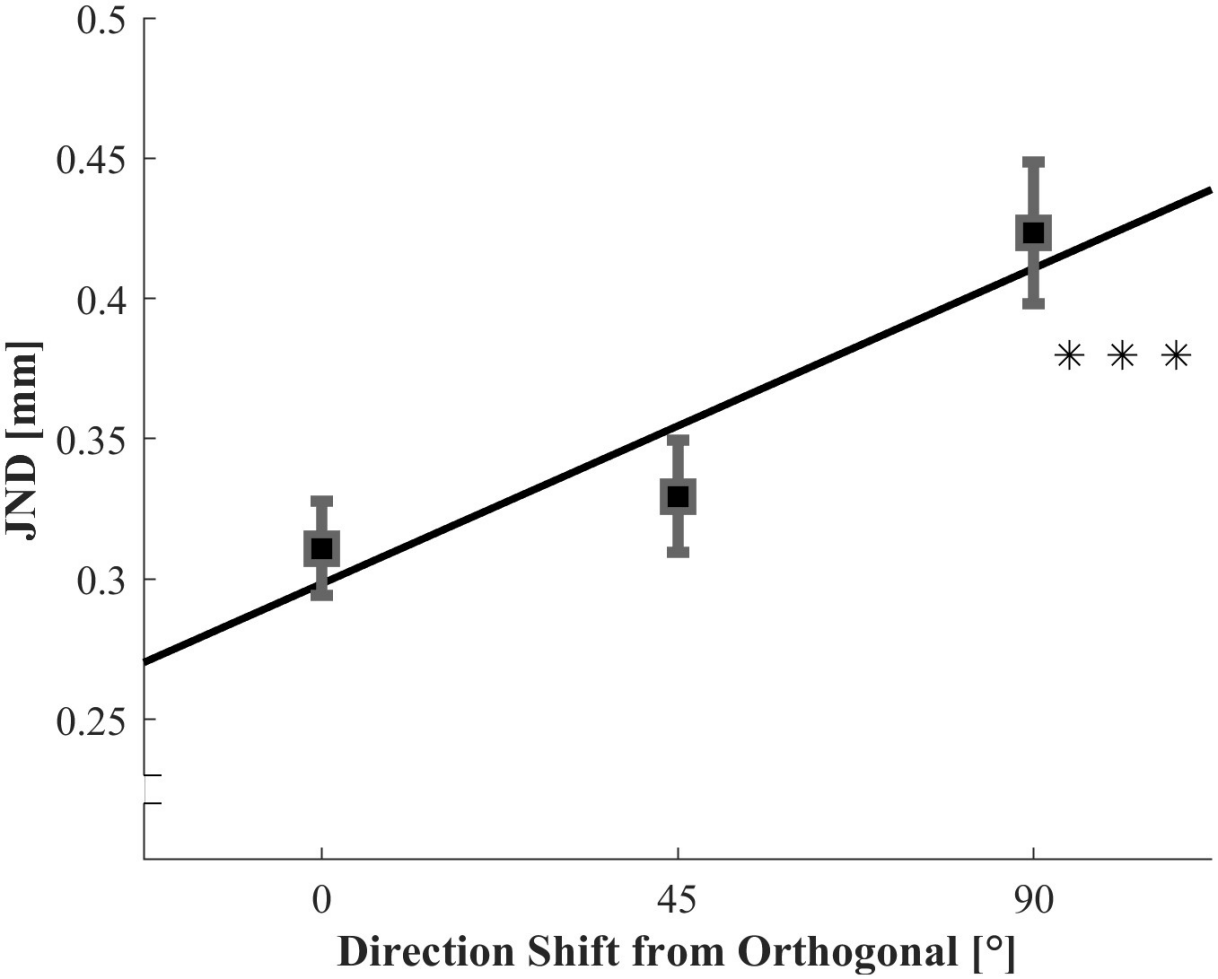
Average JNDs for the 3 conditions of movement direction shift from the texture orthogonal. Error bars are indicating within-participant standard errors [40].

Individual JNDs were entered into the ANOVA with the within participant variable Direction Shift from the texture orthogonal (0°, 45°, and 90°; depicted in Fig 3). The main effect of Direction Shift was significant, *F*(2,30) = 5.513, *p* = .009. The linear contrast analysis revealed a significant linear increase in the JNDs with larger Direction Shift, *F*(1,15) = 8.758, *p* = .005. As expected, JNDs were higher with larger Direction Shifts from the texture orthogonal. In addition, our directional a-priori hypothesis allowed for a secondary analysis through one-sided *t*-tests between individual conditions (Bonferroni-corrected alpha levels at 0.017). The JNDs were significantly higher for the 90° condition than for the 0° condition, *t*(15) = 2.959, *p* = .005. The JNDs in the 45° condition were not significantly higher than the JNDs in the 0° condition, *t*(15) = -.690, *p* = .256. They were also not significantly lower than the 90° condition, *t*(15) = 2.219, *p* = .021, but showed a trend. We conducted a sensitivity analyses with G*Power 3 [39]. The power of finding an effect of 0.15 mm (8.5% Weber fraction difference) or more was at least 96% for the Bonferroni-corrected one-sided *t*-tests (standard deviation assessed as 0.143 mm by the average standard deviation of the differences between all conditions). However, 0.15 mm is a rather large effect, comparable to difference for moving and stationary roughness discrimination in fine textures [17]. It is reasonable to expect such large effect sizes for the comparison between the extreme conditions of directional shift 0° (maximal temporal cues) and 90° (no temporal cues). The middle condition should vary by less, which is why the associated *t*-tests might not have had sufficient power to detect an effect.

### Discussion Experiment 1

In overall analysis participants were better in discriminating the spatial period of the texture as they moved more orthogonal to the texture. Although not all individual comparisons were able to confirm the effect, moving orthogonally or obliquely to the texture was or tended to be better than moving in line with the texture. The results are consistent with the prediction of a systematic monotonic increase in perceptual precision with movement directions closer to texture orthogonal, which we had made from the systematic increase of the temporal frequency of signals. A higher temporal frequency of signals likely allows for a better differentiation of textures based on temporal cues. Thus, our results support the idea that movement direction can influence perceptual precision, and that different movement directions come along with differently useful sensory signals for texture period.

In Experiment 1, we were interested in the effect of movement direction relatively to the texture orientation. However, one might wonder whether the absolute direction of the movement might also have affected texture perception. Such effects of the absolute movement direction were previously described for other tasks, such as shape perception [29]. In order to test whether the absolute movement direction might have additionally influenced the spatial period judgments, we reanalyzed the staircase data by fitting psychometric functions for all trials with the same absolute movement direction (i.e. the same exploration tunnel). Neither the points of subjective equality (PSEs), *F*(2,30) = 1.447, *p* = .251, nor the just noticeable differences (JNDs), *F*(2,30) = .891, *p* = .421, were significantly affected by the absolute direction of the movement. That is, in contrast to the results for relative movement direction we did not find evidence that also absolute movement direction considerably influenced perceptual precision nor did the different motion angles introduce a noteworthy bias in the perceived spatial period.

In optimal exploration, the systematic relationship between the movement direction relative to texture orientation and precision of perception should be exploited [23–24,28]. Some studies demonstrated that exploration movements are adjusted based on previously accumulated sensory signals—for different movement parameters during a haptic localization task [3], and for finger force during softness perception of differently compliant objects [4]. In order to test for similar mechanisms in texture perception we designed Experiment 2, in which we measure the freely chosen movement direction for texture exploration. We expect to find results complementary to Experiment 1, that is, that sensory signals for texture orientation influence movement direction.

## Experiment 2

**Fig 4:**
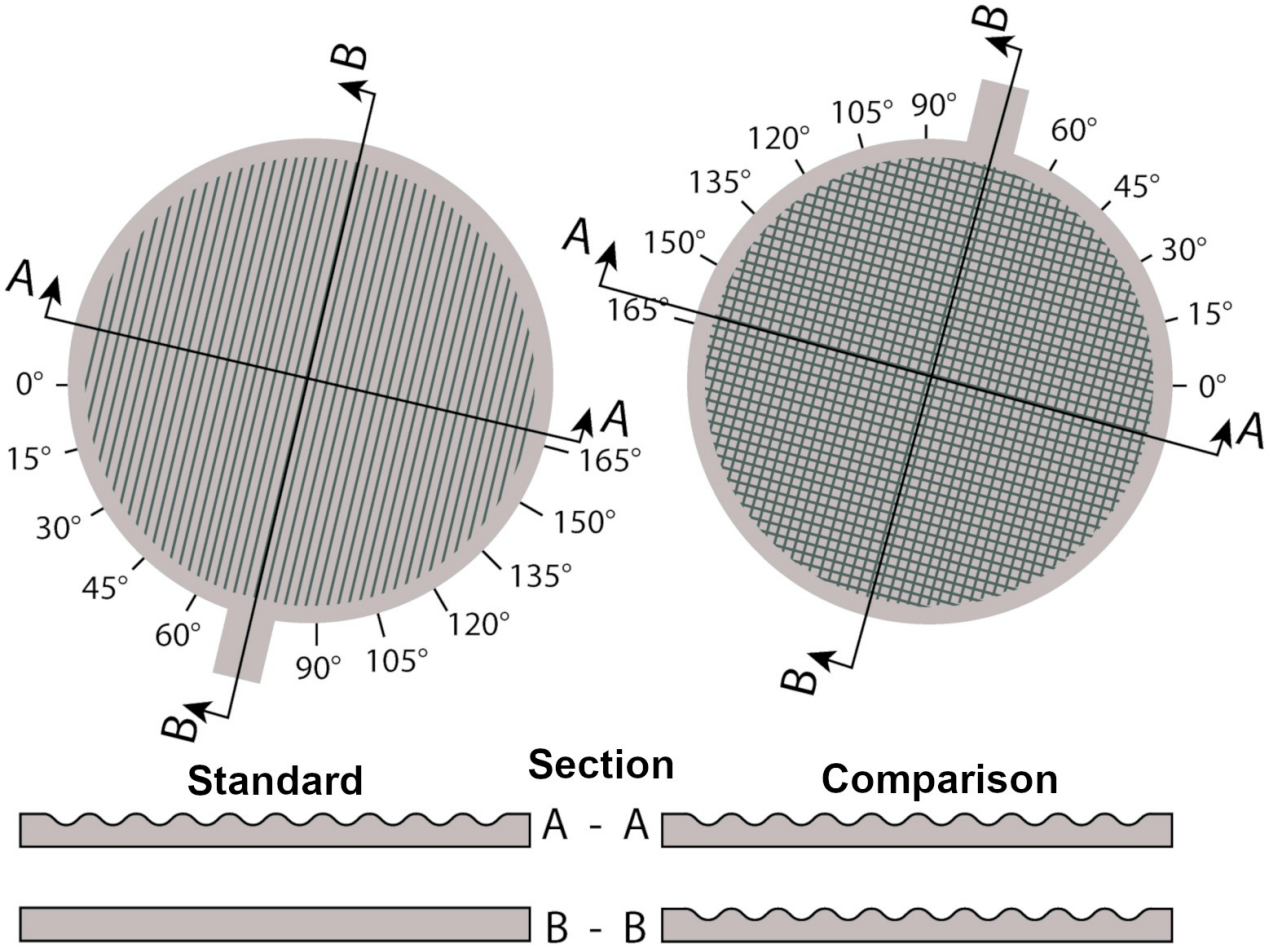
Sketch of a stimulus pair. The standard stimulus on the left is an oriented grating defined by the sine-wave function on one of the axis. The comparison stimulus on the right is a grating with no clear orientation defined by the union of two sine-wave functions on two axes. The two stimuli are depicted in the texture orientation of 75°.

In Experiment 2, we investigate movement direction in different strokes of the exploration process. We expect that movement directions are adjusted over time when doing so can improve perceptual performance, but not when there is hardly an effect of movement direction on perception. In order to test this assumption, we created two kinds of stimuli, again using 3D printing. The first type of stimuli, standard gratings, was composed of a groove pattern following the sine-wave function along one dimension, like the stimuli of Experiment 1 (periods 1.27 and 1.44 mm; Fig 4). The texture pattern of the second type of stimuli, comparison gratings, was composed of the intersections of two orthogonal sine-wave function patterns (periods: 1.02 to 1.69 mm). Thus, standards have one clear orientation, and a systematic relationship between movement direction and temporal frequency of stimulation exists: We state that for the standards, finger movements in the direction of the texture orientation generate no temporal cues to the texture period. In contrast, orthogonal movements generate optimal temporal cues by maximizing the temporal frequency of cues and, therefore, also maximizing the differences in temporal cues from different textures. For comparisons, in contrast to standards, there is not a single direction which maximizes the temporal frequency of stimulation. Movements in two orthogonal directions (0° and 90°) over comparisons provide similar temporal cues to spatial period. Participants explored one standard and one comparison stimulus grating in a trial and reported which of the two had a higher spatial-frequency. We manipulated the orientation of the stimuli in each trial, and measured movement direction for individual strokes. Participants were free to use as many strokes as they wanted. We predicted that, movements over the standard will be preferentially directed orthogonally to the texture orientation after sufficient sensory signals for orientation have been gathered. In contrast, we did not expect corresponding adjustments for the comparisons. The basic methods and a work-in-progress analysis of the raw data from the current Experiment 2 were presented in a conference paper [32]. For the sake of readability, we repeat the experimental methods in the present study. Importantly, however, the presented results are novel because raw movement data were entirely reanalyzed using improved algorithms (as described for Experiment 1).

### Methods and materials

#### Participants

Thirteen right-handed healthy participants (age range: 19–32 years; 7 females; two-point discrimination threshold 3 mm or lower) were paid for participating. Participants were naïve to the purpose of the experiment and had not participated in Experiment 1.

#### Apparatus and stimuli

The setup was identical to that used in Experiment 1, and stimuli had the same size and grip. Standard gratings were constructed exactly as for Experiment 1 (Fig 4), using two standard stimuli with the periods (P) of 1.27 mm and 1.44 mm (1 D sine-wave). In this experiment, however, we defined comparison stimuli in a way that they would not have a single clear orientation while still having spatial periods comparable to the standards. This was achieved by computing the texture height of the comparison stimuli from two overlaid sine-wave functions that were oriented perpendicular to each other. The intersection of both textures defined the comparison. A cut through two orthogonal axes of comparison stimuli would result in identical images (Fig 4), and the texture height *z* was at each point the minimum of the two sine-wave functions (2 D sine-wave; peak amplitude A = 0.3 mm):

$$z=min(\frac{1}{2}A\;sin\frac{2\pi x}{P}+\frac{1}{2}A,\frac{1}{2}A\;sin\frac{2\pi y}{P}+\frac{1}{2}A)$$ (2)

We created 5 comparison gratings with periods *P* of 1.02, 1.19, 1.35, 1.52, and 1.69 mm. For each of the two standards, we used three comparisons. Two comparisons were defined by +/- 20% of the standard’s period, because 20% corresponds to the Weber fraction in active touch ([41]; Experiment 1). The third comparison was the same stimulus for both standards (1.35 mm); it has 6% lower period than the standard of 1.44 mm and 6% higher period than the standard of 1.27 mm. Based on the stimulus construction, we defined texture orientation in standard gratings as the orientation of the parallel grooves. By definition, comparison gratings had two equal groove orientations. In the following, we will refer to one of them as the texture orientation (75° in Fig 4). It is important to note that the comparison grating had the same temporal frequency of stimulation for two movement directions, along (0°) or against (90°) its orientation. All other movement directions lowered the frequency of stimulation only moderately (< 30%). The highest deviation in temporal frequency of stimulation is produced by a movement direction of 45° to the texture orientation, which corresponds to a multiplication with sin(45°) (≈ 0.7 = -30%). Therefore, there is only a limited effect of texture orientation on the physical spatial period of comparisons, in contrast to standards.

#### Design and procedure

In each trial, a standard and a comparison stimulus were explored and participants had to judge which of the two had a higher spatial-frequency−regardless of other differences between the textures. We manipulated the orientation of the stimulus pair on the force sensor (15°, 45°, 75°, 105°, 135°, and 165°; Fig 4). We measured the movement directions over the standard and comparison gratings. Hereby, we focused on the first, middle and last stroke per stimulus, as they represent movement adjustments at different segments within the exploration process.

We used two standard stimuli paired with one of three comparisons (standard 1.27 mm with comparisons 1.02, 1.35, and 1.52 mm; standard 1.44 mm with 1.19, 1.35, and 1.69 mm). The standard was either presented at the left or the right side in order to control for potential effects of the hemispace. Both gratings of one stimulus pair were placed in the same orientation.

The focus of this experiment was on the adjustments of movements based on sensory signals gathered over the exploration process. Hence, it was essential to design this experiment in a way that encourages participants to perform a higher number of strokes over each texture. We chose a difficult perceptual task (small differences in the periods of standards and comparisons) in order to ensure that several strokes would be required to gather sufficient information for a correct response. Further, in free exploration, it is possible that participants avoid additional movement due to the associated additional movement costs. Movement costs, however, can be balanced by rewarding the performed movement [42]. Therefore, we introduced the experiment as a game and included rewards for correct responses. By giving a correct response participants could earn 10 or 100 points, which was equally distributed among all trials. Overall, the experiment consisted of 2 [standards] x 3 [comparisons] x 6 [orientations] x 2 [standard left or right] x 2 [10 or 100 points] = 144 trials. The order of the trials was randomized. Trials were presented in 3 successive blocks of 48 trials. Between two blocks, participants were instructed to take a break of at least two minutes. In total, the experiment lasted 2–3 hours. Prior to the experiment participants performed a flexible training with up to 8 trials to ensure that they understood the task.

Before each trial, the number of points corresponding to a correct response (10 or 100) was displayed on screen. When the first stimulus was displayed, a dot indicated the start position. Exploration started randomly either with the left or the right stimulus on a random position at the stimulus border (20°-350°, in steps of 30°). Participants were free to perform as many strokes and to switch as often between stimuli as they wanted. Participants received 16€ plus an additional euro for every accumulation of 500 points. Winning of this additional euro was indicated by a visual and auditory signal, which was displayed randomly 1–3 trials after the points had been accumulated. The total payment was not lower than 23€ (guessing) and not higher than 31€ (perfect performance).

#### Data analyses

Exploration movements on each stimulus were segmented into individual strokes. For the exploration of each stimulus we analyzed 3 strokes (first, middle, last). If the total number of strokes was even, the middle stroke was defined as the later one of the two possible. Strokes were segregated from the movement data as in Experiment 1 (and thus raw data was reanalyzed by improved algorithms as compared to [32]). The analysis was based only on those trials in which participants performed at least two strokes on each stimulus. When participants performed exactly two strokes, the second stroke was coded as the middle and last stroke. We aligned all stimulus orientations with an orientation of 0° in order to collapse data over trials. To do so, we rotated stroke directions by their corresponding texture orientation in opposite direction. We weighted individual strokes with their duration, as strokes had considerable differences in their duration. Based on the weighted data we calculated individual histograms of movement directions (bin size: 15°) separately for each combination of grating type and stroke (first, middle, or last). Each histogram displays which proportion of exploration time one participant moved in a specific direction. For an overall analysis, we computed an average histogram for each combination of grating type and stroke based on the individual participant analyses. For each combination of stroke (first, middle, last) and grating type (standard, comparison) circular statistics on the averaged binned data were conducted using the Matlab Circular Statistics Toolbox [43]. We performed a *V*-test, a variant of the Rayleigh test, which tests the hypothesis that the population is not distributed uniformly around the circle but has a specified mean direction (see [44]), which was 90° in our case. We applied Bonferroni-corrected alpha levels at 0.0083 (*α* = .05/6). This statistical analysis outputs *V*-values, which are higher the bigger the deviation of the empirical distribution from a uniform distribution is and the more consistent the empirical mean direction is with the predicted one. Therefore, non-significant results could either be due to a uniform distribution, or a distribution with a mean that deviates from the predicted direction of 90°. We predict that movement directions will get increasingly distributed non-uniformly over the course of the exploration of the standard stimulus. That is to say, we expect significant results for the last stroke over the standard.

### Results

#### Exploration and task performance

On average, participants spent 7.55 seconds (SD = 2.75) on the standard and performed 4.29 strokes (SD = 1.93), and they spent 7.45 seconds (SD = 2.52) on the comparison with 4.02 strokes (SD = 1.84). They switched twice between the stimuli (M = 2.05, SD = .82): once from first to the second stimulus and then once back to the first stimulus. The time spent on the stimulus did not significantly differ for the two gratings, *t*(12) = .688, *p* = .505, but participants used more strokes for the standard gratings, *t*(12) = 2.585, *p* = .024. Participants gave 59.2% correct responses on average (SD = 8%), which is significantly higher than guessing (50%), *t*(12) = 3.956, *p* = .002 (*t*-test against 50% after rationalized arcsine transformation). There was no significant difference in the arcsine-transformed percentages of correct responses between the trials with different spatial periods of the standard stimulus, *t*(12) = .024, *p* = .814. Similarly, the texture orientation did not produce a significantly non-uniform distribution of the number of correct answer, when being tested in a Rayleigh test, *R* = 0.12, *p* = .889 (means of percentage correct answers ranged between 55.1% and 64.7%).

#### Movement directions

**Fig 5:**
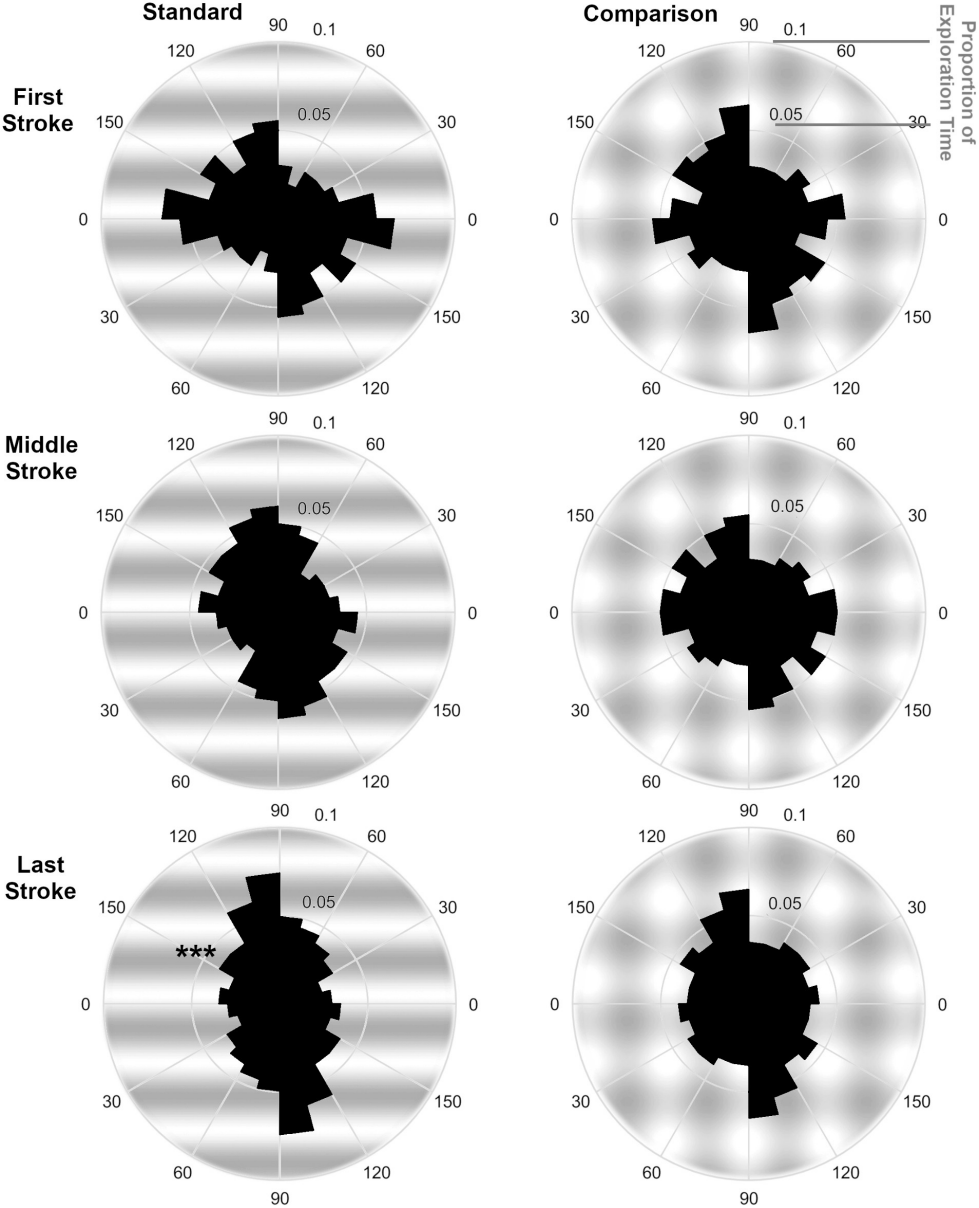
Movement direction histograms for each stroke and texture type separately including all participant data. Textures were aligned to a 0° orientation. Note, possible movement directions varied only between 0–180° and were mirrored on the lower part of each figure.

For the first, middle, and last stroke over the standard or the comparison grating we plotted the angular distributions of movement directions in Fig 5. We performed *V*-tests on each distribution testing whether it is not uniform but rather has a specified mean direction of 90° (Bonferroni-corrected alpha levels at 0.0083). In the first stroke, the *V*-tests were not significant for both gratings (standard: *V* = -8.622, *p* = .892; comparison: *V* = .167, *p* = .491). Similarly, in the middle stroke both tests did not reveal significant results, although there is a trend for the standard stimulus (standard: *V* = 10.492, *p* = .069; comparison: *V* = -2.922, *p* = .659). As predicted, participants showed a significant non-uniformity in their movement directions and moved orthogonally (90°) to the grating orientation in the last stroke over the standard, *V* = 19.425, *p* = .003. In the last stroke over the comparison, non-uniformity did not reach significance, *V* = 7.275, *p* = .152. The overall results of the *V*-tests are well reflected in the individual participant analyses when applying (Bonferroni-corrected) *V*-tests to the individual data. As expected, no participant showed more significant adjustments to the comparison than to the standard. The data of three participants had the same pattern as the average data, with an adjustment in the last stroke over the standard only. One participant adjusted in the last and middle stroke over the standard while showing no adjustment for the comparison. Four participants adjusted their middle and last stroke significantly to the standard, and the last stroke to the comparison. Five participants showed no significant adjustments to standard or comparison. For non-uniform individual distributions, the precision of the mean estimation ranged between ± 5.94° and ± 24.23° (95% confidence interval).

Additionally, in order to examine the changes in movement directions which occurred over the exploration process, we calculated the proportion of movements directed orthogonally to the texture (directions of 90° ± 15°) for the first and the last stroke. As it can be seen in Fig 5, 25% of the last strokes over standard gratings were adjusted to move approximately orthogonally to the texture orientation (directions of 90° ± 15°). In contrast, only 17% of the first movements approximated this direction. When calculated for all individual participants, the difference in percentage of movements following the 90° (± 15°) direction between the first and the last stroke over the standard is significant, *t*(12) = 4.123 *p* = .001. This is to say participants changed their movement direction significantly, from the first to the last stroke.

### Discussion Experiment 2

Experiment 2 demonstrated that participants adjust their movement direction over the course of exploration. In the first stroke, movement directions were not dependent on texture type or orientation, but rather were uniformly distributed. However, in the last stroke participants moved along the texture orthogonal for uniquely oriented textures. Movements in the last stroke were not only directed in 90° to the texture orientation, but they also were significantly adjusted from the first stroke. These results suggest that motor adjustments are based on available sensory signals for texture orientation.

One might wonder why participants’ task performance was only at about 59%. It is important to note in this regard that we purposely chose a difficult task in order to ensure that participants would perform multiple exploration movements. The difference in spatial period between the two stimuli of stimulus pair ranged from 20% (~1 Weber fraction) to 6%. Thus, performances below 70% are reasonable. Additionally, structural differences between textures (1D sine-wave vs. 2D sine-wave), can explain further performance problems. Note though, as participants were significantly better than chance, they were actually performing the task, and not guessing. We also tested whether fatigue might have decreased participants’ performance. However, a comparison of performance in the first vs. in the second half of trials, did not indicate any systematic fatigue effect (paired *t*-test after rationalized arcsine transformation for percent correct responses, *t*(12) = .636, *p* = .573).

In Experiment 3, we further test the hypothesis that motor adjustments are based on sensory signals by investigating whether sensory signals that underlie the perception of texture orientation are also used in the adjustment of movement direction. We compare the precision of the direct perception of texture orientation with that of movement adjustments to texture orientation. We use a method similar to the ‘oculometric’ functions that have been invented to compare eye movement precision to perceptual precision in vision (e.g. [33–35]). Oculometric functions mimic the construction of (perceptual) psychophysical functions by recoding eye movements into binary “motor decisions” (e.g., movement to left vs. right half of visual field). Here, we define corresponding ‘movometric’ functions for exploratory movement direction. We manipulate the spatial period of the gratings, because perceptual discrimination of gratings is known to be better for gratings with larger grooves [45], and expect that spatial period will affect perceptual and movement precision in a similar way.

## Experiment 3

Experiment 3 consisted of two parts: In each trial of the perceptual part, participants explored one oriented texture with two strokes within a limited exploration tunnel and judged the texture orientation relative to their movement direction. In the equivalent trial of the motor part, participants again performed two strokes on the same oriented texture within the limited exploration tunnel and then performed one stroke in a freely chosen direction. Here, we assessed the rotation of the freely chosen direction relative to the previous movement directions. Half of the participants started with the perceptual part and the other half with the motor part. In both experimental parts, we varied the texture orientation relatively to the exploration tunnel in the same way. Additionally, we manipulated the spatial period of the texture. The data from the perceptual part served to estimate psychometric functions on the perceived texture orientation relative to the movement direction. The data from the motor part was used to define ‘movometric’ functions on the movement adjustments made during the free stroke. In the ‘movometric’ function, the rotation of the movement direction (clockwise vs. counterclockwise) corresponds to the binary response in the psychometric function. Therefore, cumulative Gaussian functions estimating the JNDs can be fitted to the perceptual and motor response. In this way, we are able to directly compare perceptual and movement data. Because they both follow the same sensory signals, we expect that the JNDs of both the haptic orientation perception and the movement direction increase for smaller spatial period.

### Methods and materials

#### Participants

Twelve right-handed healthy participants (age 20–33 years, 8 females; two-point discrimination threshold of 3 mm or lower) entered the sample of this experiment. Participants were naïve to the purpose of the experiment and had not participated in the other two experiments.

#### Apparatus and stimuli

The apparatus was identical to that used in Experiment 1. Stimuli were defined as in Experiment 1. We used two different spatial periods *P* for the standard stimulus (1.44 and 1.86 mm). For the motor part, we additionally used 3 stimuli as the comparison stimulus (*P* = 1.27, 1.61, and 2.03 mm).

#### Procedure and design

This experiment consisted of two parts: a perceptual part and a motor part. Half of the participants started with the perceptual part and the other half with the motor part. In the perceptual part, we aimed to estimate individual psychometric functions, and in the motor part individual ‘movometric’ functions. Both parts were equivalent in the experimental design and were each preceded by 6 trials of training.

**Fig 6:**
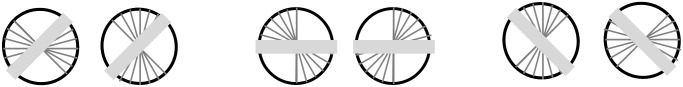
Visually displayed response options in the perceptual part of Experiment 3. Options are plotted individually for the 3 exploration tunnels (from left to right: 45°, 0°, 135°). The light grey bar depicts the exploration tunnel and the dark grey lines represent each for sample texture orientations. The left button always visualized the class of texture orientations rotated clockwise from the exploration tunnel, and thus texture orthogonals were rotated counterclockwise. The right button visualized the class, defined by counterclockwise rotation of orientation, and thus the texture orthogonal rotated clockwise from the exploration tunnel.

In the perceptual part, the task of the participant in each trial was to report the texture orientation of the standard stimulus relative to the exploration tunnel. We visualized two response options in order to get intuitive orientation judgments [46] on the upper third of the screen (Fig 6, actual size of each response option ~ 45.5 x 45.5 mm). Each of the response options stood for a class of texture orientations relative to the exploration tunnel. Response options were represented with single lines. On the left we presented the class of texture orientations, in which the texture orthogonal was rotated counterclockwise from the exploration tunnel. On the right we presented the class of texture orientations, in which the texture orthogonal was rotated clockwise from the exploration tunnel. The participant could choose one of the classes of the texture orientation by pressing virtual buttons rendered with the PHANToM. We presented texture orientation because this is intuitive for the participants to report. For our analyses, however, we recoded orientation to the corresponding texture orthogonal. As the dependent variable we measured the proportion of trials in which participants reported that the texture orthogonal was rotated counterclockwise to the exploration tunnel.

In the motor part, participants performed a two-interval forced choice (2 IFC) task judging spatial period. At the beginning of each trial, one of the comparison gratings was placed in their hands. For the haptic exploration of the comparison there were no restrictions; textures could be rotated and explored with both hands. Afterwards participants explored the standard grating. The standard grating had the same spatial period and relative orientation (of the textures orthogonal to the exploration tunnel) as in the equivalent trial of the perceptual part. However, now—after the two strokes within the exploration tunnel—the subjects were free to perform one additional stroke in any direction they wanted. We measured the movement direction in the free stroke as the dependent variable. More specifically, we looked for the proportion of trials, in which the movement direction was achieved by counterclockwise rotation from the exploration tunnel.

In each experimental part, participants explored a standard stimulus with two strokes within one of three predefined exploration tunnels (0°, 45°, or 135°). We manipulated the spatial period of the stimulus (*P* = 1.44 mm, *P* = 1.86 mm) and the rotation of the texture orthogonal relative to the exploration tunnel in 9 steps (-60°, -45°, -30°, -15°, 0°, 15°, 30°, 45°, 60°; 0° indicates the exploration direction orthogonal to the texture) following the method of constant stimuli. Additionally, the starting point within the exploration tunnel could be at either end of the tunnel which was determined randomly. In each experimental part, every combination of spatial period and relative orientation was presented 10 times, resulting in a total of 540 trials per participant (2 [spatial periods] x 9 [relative orientations] x 3 [exploration tunnels] x 10 [repetitions]). Each experimental part was subdivided into 5 blocks with 2 repetitions each and the resulting 108 trials per block were randomly ordered. Each experimental part resulted in one session of about 3 hours.

#### Data analyses

**Fig 7:**
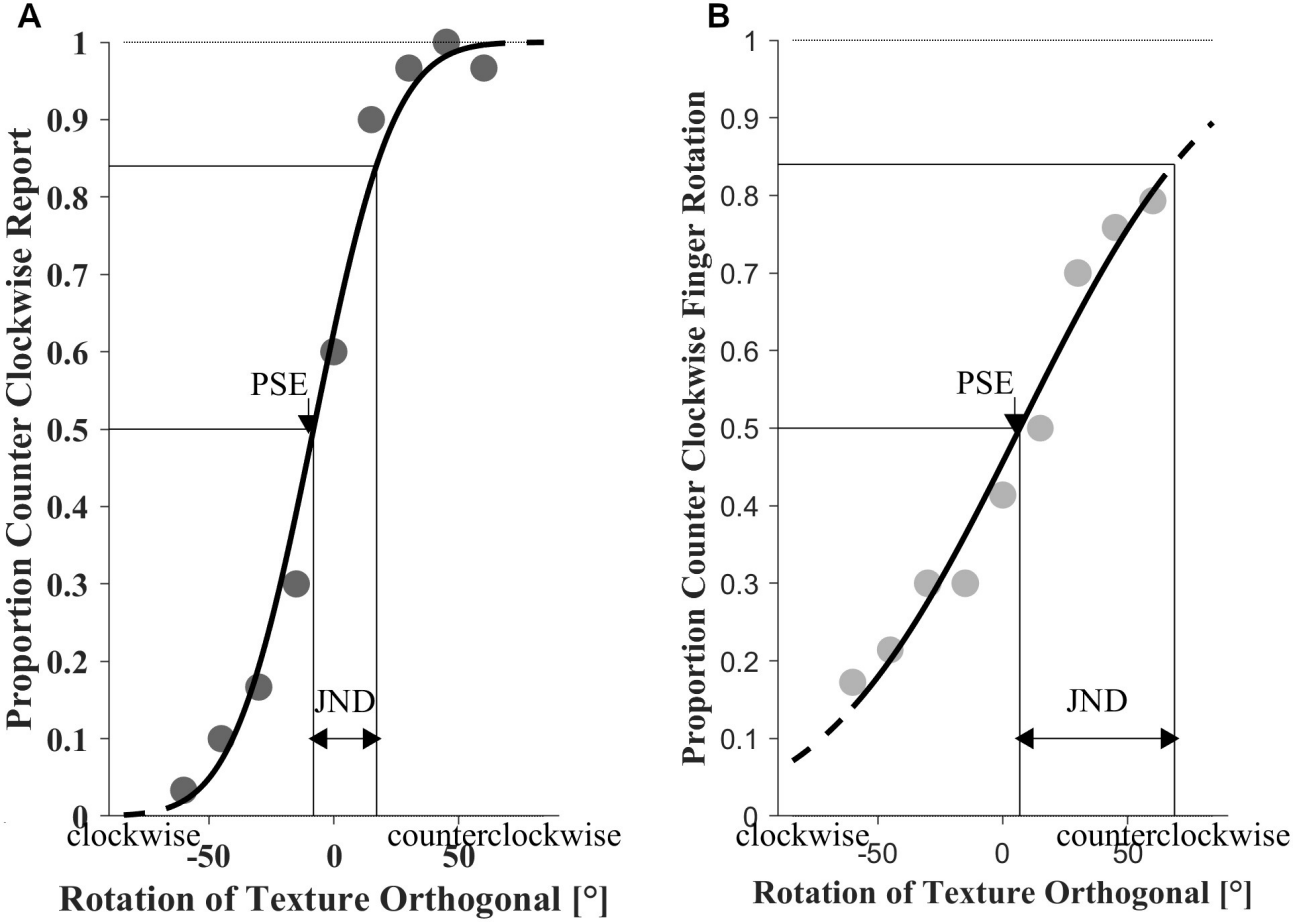
Example data of participant 9 for one standard (P = 1.86 mm). (A) psychometric curve: the proportion of trials in which the participant perceived the texture orthogonal to be rotated counterclockwise from the exploration tunnel against the actual relative rotation of the texture orthogonal. (B) ‘movometric’ curve: plotting the proportion of trials in which the participant rotated the finger counterclockwise from the exploration tunnel to perform the free stroke against the relative rotation of the texture orthogonal to the exploration tunnel.

For the perceptual part, we calculated the proportion of trials in which the participant responded that the texture orthogonal was rotated counterclockwise from the exploration tunnel as a function of the actual relative rotation of the texture orthogonal. Cumulative Gaussian functions were fit to the individual psychometric functions for each standard (see Fig 7(A) for example data). For this purpose, the psignifit4 toolbox for Matlab that implements maximum-likelihood estimation procedures was used [47]. Points of subjective equality (PSEs) were estimated by the Gaussian parameter μ and just noticeable differences (JNDs) by σ (84% discrimination thresholds). In Fig 7 the JND is indicated as the difference between the rotation values of the texture orthogonal that are associated with 50% and 84% proportions of “counterclockwise” responses.

For the motor part, the movement directions in the free stroke were analyzed as described in Experiment 1. Thereafter, we recoded movement directions into the dichotomous variable rotation from exploration tunnel (clockwise vs. counterclockwise). If a participant moves only a few degrees different from the previous stroke direction, the categorization into clockwise vs. counterclockwise rotation is straightforward. However, if a participant moves almost orthogonally to the previous stroke (around 90° / -90° rotation) the proper categorization of the underlying rotation is less clear. Therefore, trials were included only if the relative movement direction of the last stroke was rotated between—85° and + 85° from the exploration tunnel, and we were able to segregate 3 strokes (90% of trials). That is, we included data from trials with a relatively clear interpretation, which thus improved measurement precision. The total number of presented trials (270 per condition) was chosen in advance to be well above the number required for stable fitting of psychometric curves [47], so that the exclusion of some trials would not be problematic. We determined whether the executed movement direction was achieved by clockwise or counterclockwise rotation from the exploration tunnel. Rotations between 0°–85° were defined as counterclockwise rotations, whereas rotations between—85°–0° were defined as clockwise rotations.

Furthermore, we calculated the proportions of trials in which the participant rotated their finger movement counterclockwise from the exploration tunnel. To the individual ‘movometric’ functions for each standard period, we fit cumulative Gaussian functions (see Fig 7(B) for example data) using the psignifit4 toolbox for Matlab [47]. While fitting ‘movometric’ functions, we allowed for positive and negative slopes of the cumulative Gaussian, and choose the better fitting of the two curves. We used the fitting parameter ɳ as a measure of goodness-of-fit for the negative slope fit and the positive slope fit. As ɳ accounts for overdispersion and varies between 0 (no overdispersion) and 1 (high overdispersion), the fit with the lower ɳ was chosen (for details, see Schütt et al., 2016). For 18 of 24 data sets, the positive slope resulted in the better fitting curve, while for 6 data sets the negative slope provided a better fit. Because also a negative slope indicates an adjustment to the texture orientation, we will consider all the data for further analyses. However, it is important to note that the predicted main effects of the ANOVA remained significant when participants with negative slopes were excluded.

The individual psychometric and ‘movometric’ PSEs and JNDs for each standard period were entered into repeated-measures ANOVAs with the factors Mode (perception vs. movement) and Standard Period (*P* = 1.44 vs. 1.86 mm).

### Results

#### PSEs

As expected, none of the PSEs differed significantly in a single sample *t*-test against the relative Rotation of 0° (*p* ≥ .222; *P* = 1.44 mm: perception -3.9°, movement 3.2°; *P* = 1.86 mm: perception -1.7°, movement 2.4°). This result indicates that no constant biases are observed for the perceptual or the movement data. Additionally, neither any main effect nor the interaction, *F*(1,11) = .203, *p* = .661, were significant in an ANOVA with the within-participant variables Mode (perception vs. movement), *F*(1,11) = 1.432, *p* = .257, and Standard Period (1.44 vs. 1.86 mm), *F*(1,11) = .027, *p* = .871.

#### JNDs

**Fig 8:**
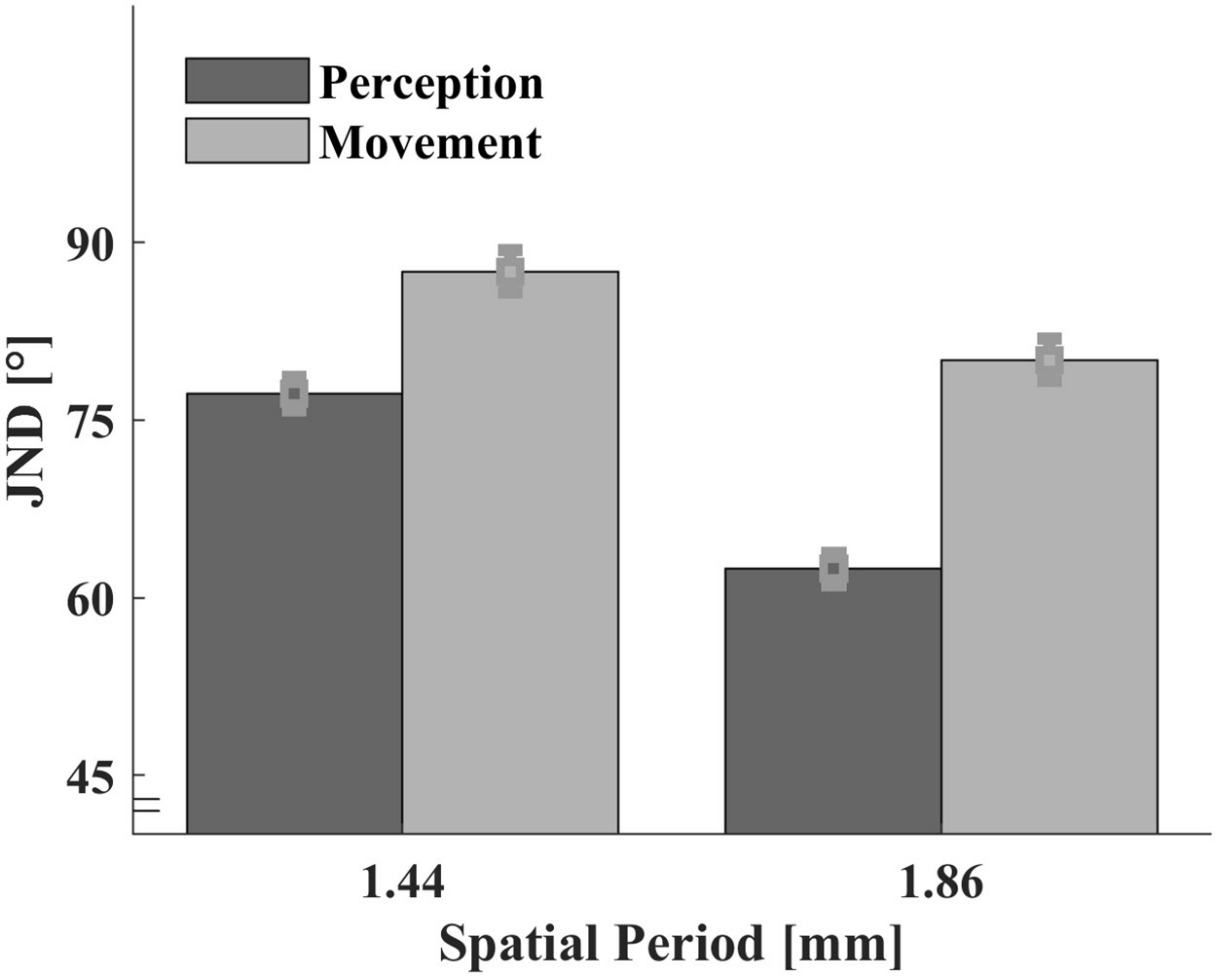
Average JNDs. JNDs from the psychometric (dark grey) and the ‘movometric’ curves (light grey) plotted with their within-participant standard errors [40].

Individual JNDs (Fig 8) entered an ANOVA with the within-participant variables Mode (perception vs. movement) and Standard Period (1.44 vs.1.86 mm). As expected, JNDs were lower for higher Spatial Period, *F*(1,11) = 34.015, *p* < .001. The JNDs were significantly lower in the perception Mode, *F*(1,11) = 5.369, *p* = .041. The interaction between the two variables did not reach significance, *F*(1,11) = 2.016, *p* = .183. The estimated statistical power of our experimental design to find an effect of at least 10° in a one-sided test is more than 90% (standard deviation assessed as 11°).

### Discussion Experiment 3

Experiment 3 carefully examined the relationship between the perceived orientation of a haptic texture and a freely chosen movement direction over the texture. Our results show that both perception and movements were influenced by spatial frequency. Participants perceived the orientation of textures with low spatial frequencies more precisely. This expands previous findings about the role of spatial period for orientation discrimination [48, 15] to active perception. Additionally, we show that movement adjustments were more pronounced in the condition of low spatial frequencies. This allows us to conclude that perception and movements are based on a similar mechanism in natural haptic exploration of surface texture.

Furthermore analyzing participants’ ‘movometric’ and psychometric curves, we found that movements were less precise than perception. This result is in line with Gegenfurtner and Franz [49], who showed that visual location perception was more precise than pointing movements to a seen location, and explained this finding by additional motor variance. Along these lines, studies on visual perception reported (at least for certain time windows) that perceptual precision was better than movement precision (e.g., [33, 50]). However, other studies on visual perception report similar perceptual and eye movement precision (e.g., [51, 33, 35]). These different findings are likely due to the complexity of the tasks, whereby less fine and more complex movements, such as the present hand movements, seem to come along with more motor variance [50–54]. However, it is important to note that in the present experiment we measured movements and perception in different experimental parts with different tasks. Although the identical standard stimulus with the identical texture orientation was presented in each given trial for both experimental parts for a specific participant, differences in the precision of movements and perception may have arisen by the fact that both were not measured in exactly the same moment in time rather than by motor variance alone. In contrast to the studies on visual perception [34, 35, 49] we, therefore, do not want to draw strong conclusion about the exact differences in the information processing. Nevertheless, given these possible differences in the measurement of movements and perception, the main effect of spatial period provides even stronger evidence for common mechanisms in orientation perception and movements in the exploration of spatial frequency.

## General discussion

This study investigated the interdependence between perception and movement directions for oriented textures. On the one hand, our results indicate that perception depends on the exact movement parameters executed: When participants followed the texture orthogonal in their movement directions more closely, they perceived the texture’s spatial period more precisely as compared to moving in line with the texture (Experiment 1, absolute movement orientation seem not to play a similar role). On the other hand, movement control depends on the sensation of texture orientation: Only after gathering sufficient sensory signals did participants adjust their movement in the direction of the texture orthogonal in free exploration (Experiment 2). In addition, sensory signals that are used to perceive texture orientation are likely also used for movement adjustment, as shown by the finding that the precision of perception and movement adjustments were influenced in the same manner by the spatial period of the stimuli (Experiment 3). Taken together, our study speaks in favor of sensorimotor control mechanisms that improve haptic perception by choosing parameters of exploratory movement on the basis of sensations.

Our results extend previous research in several ways. First, as for the long standing debate about the role of temporal cues produced by movements in texture perception (e.g., [16,17]), we provide an estimate for the advantage of movements, at least in the context of our spatial period discrimination task. We can estimate the advantage of temporal cues, when comparing JNDs measured in Experiment 1 for movement orthogonal to the texture (= optimal temporal cues) to the JNDs for movements in line with the texture (= no temporal cues). JNDs are composed of the variance for standard (σ_s_²) and the variance for the comparison stimulus (σ_c_²), which is assumed to be equal in our design (σ_c_² = σ_s_²). Under the assumption of independent percepts of comparison and standard, the JND can be directly related to the variance of the stimulus (JND_j_² = 2 σ_s_² for the condition j). The empirical JNDs indicate that the variance doubles when temporal cues are removed. In this case, the Maximum Likelihood Estimation (MLE) model of optimal integration (e.g., [55]) suggests that temporal cues are equally important and therefore should be weighted equally to spatial cues for the frequency estimation. However, it is important to note, that in contrast to other studies addressing the question on the role of temporal cues (e.g., [17,19]) we asked for a spatial frequency instead of a roughness judgment. Therefore, we cannot draw conclusions about roughness perception from our study. Nevertheless, we would suspect that when measuring roughness discriminability and manipulating directional shift of movements from texture orthogonal, results could be comparable. The reason is that we chose stimuli in a range where spatial period manipulation and roughness perception are monotonically related (see [11]). Additionally, it should be noted that previous research was also often measuring the magnitude of perceived roughness rather than its discriminability (e.g., [19]). Second, we introduced a new method, the ‘movometric’ functions, which allowed a systematic comparison between movements and perception. Such functions are not limited to the context of our task. Natural movement adjustments fordiverse exploration tasks can be used to assess the movement precision as in the present study. This only requires that movement data be converted to binary responses in order to fit ‘movometric’ functions. Future research could define such ‘movometric’ functions for movement adjustments within other exploratory procedures. For instance, indentation force is a key parameter in softness exploration [4]. Here one could fit ‘movometric’ functions to the probability that indentation force was reduced or increased as a function of stimulus softness, and then compare these to psychometric data on perceived softness.

Moreover, it is interesting to note, that not all participants moved in the way we expected. In Experiment 2, individual data for 4 out of 13 participants showed adjustments in the last stroke also over the comparison stimulus, and yet this stimulus did not have one clear orientation. While this adjustment does not seem to be very useful, it also does not harm perceptual performance. In Experiment 3, some participants adjusted their movements to the oriented textures in a way that deviated from our prediction. That is, some of the fitted psychometric curves had negative slopes. This indicates that the respective participant moved along the texture orientation rather than orthogonal to it. Nevertheless, it is important to note that even reverse adjustments indicate that these participants used sensory information to adjust their movement direction. Based on the results from Experiment 1, we could argue that these participants moved in an inefficient way. However, one possible explanation for both observations might be that, in addition to our predicted bottom-up effects, movements are also influenced by top-down effects. Thus, in Experiment 2, the 90° direction of movement over the (not uniquely oriented) comparison was possibly chosen in order to match the movement over the standard grating of the same trial. Given that the task is to compare two stimuli, moving over each of them in the same way could be a reasonable strategy. Hence, even if there is no sensory information gain to maximize (bottom-up), the task itself might influence movement control (top-down). This is in line with a recent study which showed that movement kinematics depend on both the task and the texture characteristics [56].

The task requirements might explain the unnecessary (but not inefficient) adjustments we observed in Experiment 2, but how does the task relate to the inefficient movers in Experiment 3? On first sight, the task in Experiment 3 does not seem to induce movement in line with texture orientation. However, the instruction of having only one free movement might have provoked some participants to strategize more for this task compared to more natural exploration tasks (like Experiment 2). This was also indicated by the comments of 2 observers, who reported to have chosen movements orthogonal to the previous movement. In contrast to rather natural movement planning strategies, cognitive strategic decision making seems more prone to non-optimality [57–58]. Therefore, if some participants felt the need to choose a cognitive strategy, they might have chosen the wrong one. For instance, the strategy to move orthogonal to the previous movement might seem like a good idea to collect information in the most diverse way. Taken together, some parts of the data might be due to task induced top-down influence on movement control. However, we suggest that these task effects act in addition to our proposed sensorimotor processes and are not an alternative to it. We base this assumption on the fact that these movement effects are represented in some individual participants, not the average data across participants. Hence, they also do not devaluate our significant findings. Further studies might systematically investigate the importance of top-down influences for movement control in haptic exploration.

Overall, we presented evidence that perception and movement are highly interdependent for the exploration of oriented textures. Sensory information about texture orientation is used to adjust movement directions towards the texture orthogonal. As a consequence, optimal sensory information about the structure of the texture can be extracted and used for the perceptual task. Interestingly, this co-influence happens, although it was shown that textural orientation and structure information are not processed within the same pathway [15, 59–60]. By introducing a method, which allows for a direct comparison between perception and movement control, we were able to demonstrate that shared sensory information is supplied to both systems. Future studies can apply our method to study other perceptual dimensions, which will help to understand the interplay between sensory and motor processes in general.
